# IL-18 serves as a main effector of CAF-derived METTL3 against immunosuppression of NSCLC via driving NF-κB pathway

**DOI:** 10.1080/15592294.2023.2265625

**Published:** 2023-10-23

**Authors:** Li Xu, Kang Li, Jia Li, Fang Xu, Shuzhi Liang, Yi Kong, Bolin Chen

**Affiliations:** The Second Department of Thoracic Oncology, Hunan Cancer Hospital/The Affiliated Cancer Hospital of Xiangya School of Medicine, Central South University, Changsha, Hunan Province, P.R. China

**Keywords:** METTL3, m^6^A, immunosuppression, IL-18, NSCLC

## Abstract

**Background:** N6-methyladenosine (m^6^A) is the most abundant modification in eukaryotic mRNA. However, its role in non-small cell lung cancer (NSCLC) has not been completely elucidated.

**Objective:** To explore whether methyltransferase like 3 (METTL3) in cancer associated fibroblasts (CAFs) affects the secretion of IL-18, which drives NSCLC cells to regulate PD-L1-mediated immunosuppression via the nuclear factor kappa B (NF-κB) pathway.

**Methods:** Histopathological features of NSCLC tissues were identified by H&E and IHC staining. The levels of m^6^A writers (METTL3), IL-18 and NF-κB pathway related genes were assessed. The quantity of CD8+ T cells was evaluated by flow cytometry (FCM). The direct binding relationship between METTL3 and IL-18 mRNA was detected by RIP assay and RNA pulldown and confirmed by dual – luciferase reporter assay. The level of RNA m^6^A was detected by RNA m^6^A dot blot and meRIP assays. A heterotopic implantation model of NSCLC was established in NOD-SCID mice for further explore the effect of CAF derived METTL3 on immunosuppression of NSCLC *in* *vivo*.

**Results:** Our results illustrated that METTL3 was down-regulated in CAFs, and CAF derived METTL3 alleviated PD-L1-mediated immunosuppression of NSCLC through IL-18. Subsequently, we found that IL-18 was main effector of CAF-derived METTL3 against immunosuppression of NSCLC, and IL-18 accelerated immunosuppression of NSCLC by driving NF-κB pathway. *In vivo*, METTL3 knockdown-derived CAFs accelerated immunosuppression of NSCLC.

**Conclusion:** IL-18 served as a main effector of CAF-derived METTL3 against immunosuppression of NSCLC via driving NF-κB pathway.

## Introduction

Lung cancer is the most common cancer, which is one of the main causes of tumour-related death. Statistically, there are more than 2 million new cases of lung cancer worldwide, of which nearly 1.7 million die every year [1]. Lung cancer includes NSCLC and small cell lung cancer (SCLC), NSCLC accounts for 85% of all cases and has a high incidence [2]. Due to the lack of effective diagnostic tools, many patients are only diagnosed at a late stage, missing the best time for intervention [3]. At present, surgical treatment is one of the main treatment strategies, but the 5-year survival rate is not ideal due to the easy recurrence and rapid metastasis of NSCLC, which is less than 20% [4]. Therefore, further elucidation of the molecular mechanism of NSCLC and the pathogenesis of NSCLC will help to provide early treatment and obtain a better prognosis. CAFs is an active fibroblast in tumour tissue, which is also the main component of tumour microenvironment, and CAFs accelerates the malignant progression of tumours by producing cytokines or growth factors [5]. CAFs regulate the inflammatory microenvironment by promoting the expression of inflammatory cytokines such as IL-1β, IL-6 and IL-8 in NSCLC [6]. Tumour cells evade the immune surveillance by up-regulating surface expression of programmed death ligand 1 (PD-L1), which interacts with PD-1 on T cells to elicit the immune checkpoint response [7]. PD-L1 is encoded by the 8-exon CD274 gene on the 9p24.1 chromosome. In tumour microenvironment, PD-L1 plays an important checkpoint role in T cell-mediated anti-tumour immune response via regulating the downstream signal of T cell receptor (TCR) [8]. The high expression of PD-L1 will help tumour cells to form immunosuppression, which will bring some challenges to the treatment of tumours [9]. Impressive clinical successes have been observed in the use of PD-1 checkpoints to block immunotherapy in a variety of cancers’ treatment, including NSCLC [10]. However, the limited response of most patients treated with anti-PD-1 antibodies remains a challenge, and a better understanding of the molecular mechanisms underlying restrictive immunotherapy is required. N6-methyladenosine (m^6^A) is the most abundant mRNA modification in mammals, which is regulated by the balance activity of m^6^A ‘writer’ and ‘eraser’ proteins [11]. m^6^A is installed by RNA methyltransferase complex, m^6^A writer, which is composed of methyltransferase like 3 (METTL3), METTL14 and WTAP, etc. Among them, METTL3 plays an important role in various cancer progression [12]. METTL3 regulated m^6^A modification of IGF2BP2 to facilitate colorectal carcinoma progression [13]. METTL3 regulates bladder cancer immune escape by regulating m^6^A modification of PD-L1 mRNA [14]. YTH N6-methyladenosine RNA-binding protein 2 (YTHDF2) destabilizes m^6^A-modified RNA via serval different mechanisms, including de-adenylation by the CCR4-NOT complex, and endoribonucleolytic cleavage by HRSP12-RNase P/MRP [15,16]. Besides, YTHDF2, an m^6^A reader, was reported to selectively bind to m^6^A sites to regulate mRNA degradation. Accumulating evidence has shown that it is involved in tumorigenesis [17]. However, the role of METTL3/YTHDF2 axis in CAFs and its specific molecular mechanism in NSCLC progression remains unclear.

Interleukin 18 (IL-18) is located on the chromosome of 11q22.2-q22.3 and belongs to interleukin-1 superfamily [18]. IL-18 is expressed and secreted by immune cells, which plays a key role in the regulation of inflammation and immune response [19]. Early evidence suggested that IL-18 has anticancer effect on tumour, which stimulated the cytotoxicity of natural killer cells (NKs) and enhanced the immune response of CD8^+^T lymphocytes (CD8^+^T) [20]. However, studies have shown that IL-18 was high expression in the serum of NSCLC patients, and which was positively correlated with PD-L1, and IL-18 induces PD-L1 expression and induced PD-L1-dependent immunosuppression [21]. Then, SRAMP website (http://www.cuilab.cn/sramp/) prediction analysis showed that there were seven m^6^A modification sites in GenBank: AF191088.1 of IL-18 and two m^6^A modification sites in GenBank: AF226165.1. Therefore, we speculated that METTL3 mediates IL-18 mRNA m^6^A adenosine methylation to inhibit its expression. NF-κB is a transcription factor implicated in inflammation, immunity, and cancer. NF-κB signalling is activated by oxidative stress, DNA damage, necrotic cell products, bacterial infections, and pro-inflammatory cytokines [22]. Interestingly, NF-κB regulates the level of CD274 (encoding PD-L1) in various cancer types, and p65 enhances the transcriptional activity of NF-κB and p65 facilitates the expression of PD-L1 through binding to PD-L1 promoter [23]. Furthermore, it is reported that IL-18 stimulates the activation of NF-κB to activate NF-κB pathway in acute pancreatitis [24]. However, the interaction between IL-18 and NF-κB pathway in NSCLC immunosuppression remains unclear.

Summary, we hypothesized that METTL3 with low expression in CAFs against immunosuppression of NSCLC via up-regulating IL-18, resulting in driving NF-κB pathway in NSCLC cells. We aimed to clarify the potential molecular mechanism of CAF-derived METTL3 regulating immunosuppression of NSCLC.

## Materials and methods

### Specimen

From May 2020 to June 2021, after receiving the written informed consent, Hunan Cancer Hospital collected the information of 30 NSCLC patients (25–45 years old, including 16 males and 14 females), and collected surgical tumour samples and adjacent non-tumour tissues. 30 selected patients did not receive chemotherapy and/or radiotherapy. The samples were immediately frozen in liquid nitrogen for verification. This study was approved by the Ethics Committee of Hunan Cancer Hospital and obtained the written informed consent of all patients (No. SBQLL-2021-215). All the experiments were carried out according to approved guidelines.

### Cell lines and culture

HEK293T cell lines and NSCLC cell lines including A549 and H1650 cells were provided by American Type Culture Collection (ATCC, Manassas, VA, USA), cultivated within Corning™ DMEM Medium (11965092, Invitrogen, California, USA) that contained 10% foetal bovine serum (FBS, Gibico, NY, USA) and incubated within the humid incubator under 37°C and 5% CO_2_ conditions.

### Cell treatment

sh-METTL3, sh-YTHDF2, sh-IL-18 and its negative control sh-NC were transfected into CAFs for METTL3, YTHDF2 or IL-18 knockdown. Subsequently, the full-length sequences of METTL3 or IL-18 were inserted into pcDNA3.1 (Invitrogen, California, USA) for METTL3 or IL-18 overexpression, their negative control pcDNA3.1-NC was obtained in the same way. Lipofectamine™ 3000 Transfection Reagent (Invitrogen) was used to transfect corresponding plasmids into CAFs or NSCLC cells. Subsequently, A549 or H1650 cells were randomly divided into six groups. Control, A549 or H1650 cells were untreated; CAF-CM group, A549 or H1650 cells were co-cultured with CAFs-derived culture medium (CM); CAF-sh-NC-CM group, A549 or H1650 cells were co-cultured with CM from CAFs which transfected with sh-NC; CAF-sh-METTL3-CM group, A549 or H1650 cells were co-cultured with CM from CAFs which transfected with sh-METTL3; CAF-oe-NC-CM group, A549 or H1650 cells were co-cultured with CM from CAFs which transfected with pcDNA3.1-NC; CAF-oe-METTL3-CM group, A549 or H1650 cells were co-cultured with CM from CAFs which were transfected with pcDNA3.1-METTL3. Furthermore, CD8^+^T cells were co-cultured with each group of NSCLC cells (A549 or H1650), respectively, and the cytotoxicity of CD8^+^ T cells was evaluated. This study was approved by the Ethics Committee of Hunan Cancer Hospital and all the experiments were carried out according to approved guidelines (2021–031). Besides, BAY-11-7508, served as the inhibitor of NF-κB signal pathway was purchased from MCE (Shanghai, China), and IL-18BP was also was purchased from MCE.

### Animal model

A heterotopic implantation model of NSCLC was established in NOD-SCID mice (20–40 g) for 4–6 weeks. Firstly, the full-length METTL3 cDNA was cloned into pEGFP-C1 vector (Addgene Company, USA) and the recombinant pEGFP-METTL3 vector was constructed. pMD2G, psPAX2 and pLKO.1 plasmid packaging system was used to produce lentivirus particles (Addgene Company, Watertown, MA, USA). Culture supernatants containing lentivirus particles were collected 48 h after transfection. CAFs was infected with lentivirus particles to construct the METTL3 knockdown-CAFs (shMETTL3-CAFs). Subsequently, A549 or H1650 cells (1 × 10^6^ cells per mouse) which were co-cultured with CAFs were injected -SCID mice at 1:1 for NSCLC model establishment. NOD-SCID mice were randomly divided into five groups, control group, CAFs group, mice were injected with NSCLC cells (A549 and H1650 cells, 1 × 10^6^ cells per mouse) co-cultured with CAFs; CAF-sh-NC group, mice were injected with NSCLC cells (1 × 10^6^ cells per mouse) co-cultured with -shNC-CAFs; CAF-sh-METTL3 group, mice were injected with NSCLC cells (1 × 10^6^ cells per mouse) co-cultured with shMETTL3-CAFs; CAFs-shMETTL3 plus BAY11–7085, mice were injected with NSCLC cells (1 × 10^6^ cells per mouse) co-cultured with shMETTL3-CAFs and furtherly injected with BAY11–7085 which served as the inhibitor of NF-κB signal pathway. PBMCs from healthy donors were activated and expanded. The day before NSCLC cell injection, PBMC (i.v. 1 × 10^7^ cells) was adoptively transferred to NOD-SCID mice via the tail vein. Mice were injected continuously for 5 weeks, and the tumour size was recorded every day. After 5 weeks, mice were sacrificed by cervical dislocation and the tumour was removed for statistics of tumour size, weight and volume.

### CD8^+^T isolation and identification

After the dead cells were removed from the filter by stromal cell suspension, CD8^+^T cells were selected and isolated using the negative magnetic beads of CD8^+^T cell separation kit (Miltenyi Biotec). In addition, anti-fibroblast microspheres (Miltenyi Biotec) were added with the microspheres attached to the kit to ensure that the interstitial fibroblasts in the mixed cell suspension were depleted. After two rounds of negative selection, the purity of CD8^+^T cell population was more than 90%. The average total number of CD8^+^T cells recovered per gram of tissue was 2 × 10^5^. After isolation, the purified CD8^+^T cells were suspended in X-VIVO 15 Media (Lonza, Walkersville, MD) containing 10% carbon stripped human AB serum. (Valley Biomedical, Winchester, VA).

### The cytotoxicity of CD8^+^T

Firstly, CD8^+^T were co-cultured with CAFs or METTL3 knockdown/overexpression-CAFs for 72 h. What we used here was direct co-culture. Subsequently, the cells were collected and homogenized by ultrasonic cell crusher, and the supernatant was collected after centrifugation 10 min. The cytotoxicity of CD8^+^T were detected by LDH Cytotoxicity Assay Kit (Beyotime, Nanjing, China), according to the instructions.

### CAFs isolation and identification

**Figure 1.**
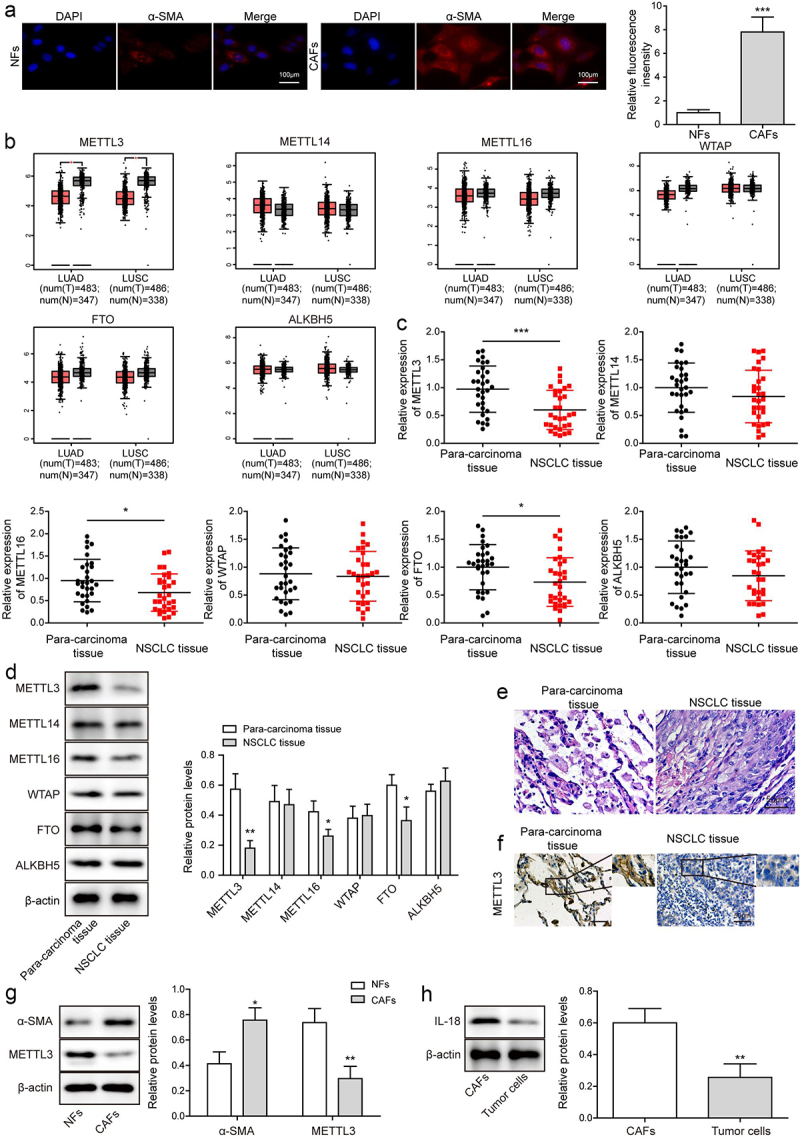
METTL3 was down-regulated in CAFs. CAFs were separated from NSCLC tissues, and NFs were separated from para-carcinoma tissues. a, the morphology of CAFs and NFs was identified by IF. Scale bar = 100 μm. b, the level of METTL3, METTL16, METTL14, WTAP, FTO and ALKBH5 were analysed by TCGA database. c, m^6^A writers’ levels, including METTL14, METTL3, WTAP, METTL16, FTO and ALKBH5 were detected by qRT-PCR. d, m^6^A writers’ levels, including METTL14, METTL3, WTAP, FTO, ALKBH5 and METTL16 were detected by Western blot. e, histopathological feature of NSCLC tissues and para-carcinoma tissues was evaluated by H&E staining. Scale bar = 50 μm. f, METTL3 level in NSCLC tissues and para-carcinoma tissues was analysed by IHC. Scale bar = 50 μm. g, α-SMA and METTL3 level in CAFs and NFs. h, IL-18 expression in CAFs and tumour cells of NSCLC was detected. All data were shown as mean ± SD. *n* = 3 per group. *P < 0.05, **P < 0.01, ***P < 0.001.

CAFs and primary cancer cells were isolated from NSCLC tumour tissues by primary culture, while NFs were derived from the paired adjacent normal tissues, CAFs were identified by the presence of CAF-specific markers (α-SMA), according to previous reports [25], and CAFs were identified by IF assay (Figure 1a). In detail, CAFs was immobilized in methanol for 4 min and placed in acetone for 3 min, and washed with phosphate-buffered saline (PBS) three times. Subsequently, CAFs were permeated with 0.2% Triton X-100 in PBS for 5 min, and the potential non-specific binding sites were blocked by 1% IgG-free bovine serum albumin at room temperature. Then CAFs was incubated overnight with anti-α-SMA antibody (ab244177, Abcam, Cambridge, UK) at 4°C. The second day, CAFs were washed with PBS three times and incubated with secondary antibodies at room temperature for 1 h. Finally, CAFs were washed with PBS three times and were observed under a Nikon Instruments C2 Plus Confocal Microscope (Nikon Instruments, Melville, NY, USA). For the collection of CAF-CM, the culture medium (CM) was collected and centrifuged to remove cell pellets. A549 and H1650 cells were co-cultured with the CAF-CM and then were subjected for cytological experiments.

### qRT-PCR and mRNA stability analysis

Total RNA was extracted with TRIzol reagent, One-Step SYBR Prime Script PLUS RT-PCR kit was used to detect the levels of METTL3, METTL14, METTL16, WTAP, FTO, ALKBH5, IL-18, and YTHDF2 genes. GAPDH was used as the endogenous control in data analysis. Finally, fold changes were calculated using the 2^−ΔΔCt^ method. The whole process was repeated three times. The primers used in this study were shown in Table 1. Besides, CAFs were transfected with shYTHDF2 for 48 h, and then treated with 5 mM Actinomycin D (HY-17559, MCE), finally harvested at the indicated time points (0, 2, 4, 6 and 8 h) to evaluate the remaining IL-18 mRNA.

**Table 1.** Primer sequences.

Primer name	Primer sequences
F- METTL3	5′-GAGTGCATGAAAGCCAGTGA-3′
*R*- METTL3	5′-CTGGAATCACCTCCGACACT-3′
F- METTL14	5′-AGGGGTTGGACCTTGGAAGA-3′
*R*- METTL14	5′-GAAGTCCCCGTCTGTGCTAC-3′
F- METTL16	5′-GGCAGAAGGAGGTGAATTAGAG-3′
*R*- METTL16	5′-TTCCCAGCATGCAGCTATAC-3′
F-WTAP	5′-TGGCAGAGGAGGTAGTGGTT-3′
R-WTAP	5′-GGGAACCCACAGTTCGATTA-3′
F-FTO	5′-ACCTCCAGCATTAGATTC-3′
R-FTO	5′-GAAACTACCGCATTTACC-3′
F-ALKBH5	5′-TGAGCACAGTCACGCTTCCC-3′
R-ALKBH5	5′-TCCGTGTCCTTCTTTAGCGACTC-3′
F-YTHDF2	5′-AGCCCCACTTCCTACCAGATG-3′
R-YTHDF2	5′-TGAGAACTGTTATTTCCCCATGC-3′
F-IL-18	5′-TGGCTGCTGAACCAGTAGAA-3′
R-IL-18	5′-ATAGAGGCCGATTTCCTTGG-3′
F-GAPDH	5′-CCAGGTGGTCTCCTCTGA-3′
*R*- GAPDH	5′-GCTGTAGCCAAATCGTTGT-3′

### Western blot

A549 or H1650 cells were lysed. Then, the proteins were quantified using a BCA assay (#23225, Thermo Scientific). Subsequently, an equal amount of protein samples was subjected to sodium dodecyl sulphate-polyacrylamide gel electrophoresis (SDS-PAGE) and electro-transferred to 0.22 μm polyvinylidene difluoride membranes (PVDF, Millipore, HATF09025). The membranes were blocked with 5% BSA and incubated rabbit anti-PD-L1 (ab205921, Abcam, Cambridge, UK), anti-IL-18 (ab243091, Abcam), anti-p65 (ab32536, Abcam), anti-p-p65 (ab31624, Abcam), anti-METTL3 (ab195352, Abcam), anti-p-IκBα (#2859, Cell Signaling), anti-IκBα (#9242, Cell Signaling), anti-YTHDF2 (#80014, Cell Signaling), anti-METT16 (#17676, Cell Signaling), anti-WTAP (#56501, Cell Signaling), anti-FTO (#14386, Cell Signaling), anti-ALKBH5 (#80283, Cell Signaling), anti-METTL14 (#51104, Cell Signaling) and anti-α-SMA (#14968, Cell Signaling) antibodies overnight at 4°C.β-actin (ab8226, Abcam) was used as the endogenous control. On the second day, the membranes were then incubated with a secondary antibody (Jackson, 1:10000) for 1 h at room temperature followed by three 10 min washes in TBST. The bands were visualized using an enhanced chemiluminescence (ECL) Detection Kit (E-IR-R308, Elabscience). Data were quantified using the ImageJ Launcher software (National Institutes of Health).

### RIP assay

RIP analysis was performed in CAFs using the Magna RIP RNA-binding protein immunoprecipitation kit (Millipore, Bedford, MA, United States) and antibody against YTHDF2 (ab220163, Abcam). Simply, incubated the complex of magnetic beads and antibody with the cell lysate at 4°C for more than 12 h, then elute, extract, and purify the RNA. Reverse transcription-quantitative polymerase chain reaction to measure YTHDF2 level.

### RNA pulldown

BersinBio RNA pull-down kit (BersinBio, Guangzhou, China) was used to performed RNA pulldown. Targeted IL-18 biotin labelled probes were designed and synthesized by GenePharma (Shanghai, China). RNA-protein complexes were formed by incubating specific probes with cell lysates. Then, the complex was separated by streptavidin conjugated magnetic beads. After separation by 10% polyacrylamide gel electrophoresis (PAGE), Western blot was performed to measure YTHDF2 (#80014, Cell Signaling) level.

### H&E staining

H&E staining was used to evaluate histopathological features of NSCLC tissues. In short, tissues were cut into 5 μm thick blocks and fixed overnight with 10% neutral buffer formalin at 4°C. The next day, the tissue sections were dehydrated, washed and embedded in paraffin with graded ethanol series. Subsequently, the sections were stained with haematoxylin for 10 min and eosin for 5 min. Finally, tissue sections were imaged with an optical microscope (BD Pharmingen, San Diego, California).

### Immunohistochemistry (IHC)

Firstly, NSCLC tissues and mouse tumour tissues were fixed in 4% paraformaldehyde, then were paraffin-embedded and sectioned. Slides were incubated with METTL3 (#86132, Cell Signaling) or CD8 (#70306, Cell Signaling) primary antibodies at 4°C overnight, incubated with a secondary antibody, and then the chromogenic reaction was conducted with 3, 3-diaminobenzidine (DA1015, Solarbio) and counterstained with haematoxylin (G1080, Solarbio, Beijing China) according to the corresponding manufacturer’s instructions. Images were captured using the Nikon microscope.

### Flow cytometry (FCM)

Firstly, A549 and H1650 cells were cocultured with CAF-CM or METTL3 knockdown or overexpression-CAF-CM for 72 h. Next, at least 10,000 live cells were collected, then cells coupled with fluorescein isothiocyanate (FITC) was incubated with anti-PD-L1 (ab205921, Abcam) for 30 min, and analysed on the flow cytometer (Beckman, CytoFLEX, Brea, CA, USA). Experimental data were analysed using Flow Jo 7.6 and Modifit software.

### ELISA

Firstly, CD8^+^T were co-cultured with NSCLC cells (A549 and H1650), which pretreated with CAF-CM or METTL3 knockdown or overexpression-CAF-CM for 72 h. Subsequently, the cells were collected and homogenized by ultrasonic cell crusher, and the supernatant was collected after centrifugation 10 min. Then the levels of granzyme B and perforin were detected by Human GzmB (Granzyme B) ELISA Kit (E-EL-H1617c, Elabscience, Beijing, China) and Human PRF1 (Perforin 1) ELISA Kit (E-EL-H1123c, Elabscience), the level of IL-18 was detected by Human IL-18 ELISA Kit (E-EL-H0253c, Elabscience), according to instructions.

### METTL3 target selection and dual luciferase reporter assay

The m^6^A motif sites of IL-18 coding region were furtherly predicted by SRAMP website, m^6^A motif sites in the IL-18 coding region and the map of the secondary structure of RNA at 180 points was predicted by the SRAMP website (http://www.cuilab.cn/sramp/), and the physical interaction between METTL3 and IL-18 was confirmed by dual-luciferase reporter assay. In detail, wild-type sequences and mutant sequences (WT-IL-18, MUT-IL-18) were designed and synthesized based on the predicted binding sites. The potential binding sequences of METTL3 on the IL-18 mRNA were mutated using the QuikChange™ Site-Directed Mutagenesis Kit (Stratagene, La Jolla, CA, United States). Sequences of WT-IL-18, MUT-IL-18 were inserted into luciferase reporter vector (pGL3-Basic). Subsequently, HEK293T cells were co-transfected with IL-18-WT, IL-18-MUT, sh-METTL3 and simulated NC by Lipofectamine™3000 (Takara, Dalian, China). Luciferase activity was detected 48 h after transfection.

### RNA m^6^A dot blot assays

Poly (A) GE Healthcare + RNAs was heated at 65°C for 5 min and then transferred to nitrocellulose (NC) membrane (Amersham, GE Healthcare, USA). Subsequently, NC membranes were incubated with m^6^A antibody (ab284130, Abcam) overnight at 4°C. Then NC membrane was incubated with goat anti-mouse IgG bound by HRP (ab150077, Abcam) for 1 h. Finally, the membranes were visualized by the ECL Detection Kit (E-IR-R308, Elabscience), and data were quantified using the ImageJ Launcher software (National Institutes of Health)….

### MeRIP qPCR assay

Total RNA was extracted from A549 or H1650 cells transfected with sh-METTL3 and treated with DNase (Sigma) to remove genomic DNA. Immunoprecipitation was performed by incubating RNA with m^6^A primary antibody (ab286164, Abcam) using the GenSeq® m^6^A MeRIP Kit (GS-ET-001, Cloud-seq, Shanghai, China). Finally, the mRNA of the enriched m^6^A-modified IL-8 was detected by qRT-PCR.

### Statistical analysis

The mean ± standard deviation (SD) represents data from three independent experiments. Statistical analysis of all data was performed by using GraphPad Prism 5.0 Software (GraphPad Software, Inc.). One-way ANOVA or Student t test was used in the comparison between the two groups, and the Tukey post-test was used in the multi-group comparison. Correlations between genes were analysed using Pearson’s correlation coefficient. When *P* < 0.05, the difference is statistically significant.

## Results

### METTL3 was down-regulated in CAFs

Some studies have shown that human METTL3 was a kind of m^6^A methyltransferase [26]. To explore METTL3 level in CAFs and NFs, firstly, CAFs were separated from NSCLC tissues, and NFs were separated from para-carcinoma tissues. Under the microscope, we observed that the morphology of the cells separated from NSCLC tissues changed from star-shaped or polygonal to flat and long fusiform, the cytoplasmic synapses decreased obviously, and the cells grew bipolar, and fluorescence of α-SMA which serves as CAF biomarker was observed (Figure 1a), indicating CAFs were isolated successfully [27]. Subsequently, GEIPA database (http://gepia.cancer-pku.cn/) was used to analyse the levels of METTL3, METTL14, METTL16, WTAP, FTO and ALKBH5 genes. Analysis showed that METTL3 which served as the m^6^A writers was significantly down-regulated (Figure 1b). Consistently, similar trend was observed by qRT-PCR and Western blot analysis (Figure 1c,d).Besides, the level of METTL16 and FTO were slightly down-regulated, while the level of METTL14, WTAP and ALKBH5 had no change, which was demonstrated in Figure 1c,d. Next, NSCLC tissue was identified by H&E staining (Figure 1e), and IHC analysis showed that METTL3 was significantly downregulated in NSCLC tissue (Figure 1f). Besides, as showing in Figure 1g, compared with that in NFs, METTL3 was downregulated in CAFs. Furtherly, IL-18 expression in CAFs and tumour cells of NSCLC was detected. Results indicated that compared with that in tumour cells of NSCLC, IL-18 was significantly up-regulated in CAFs (Figure 1gh).

### CAF derived METTL3 alleviated PD-L1-mediated immunosuppression of NSCLC

**Figure 2.**
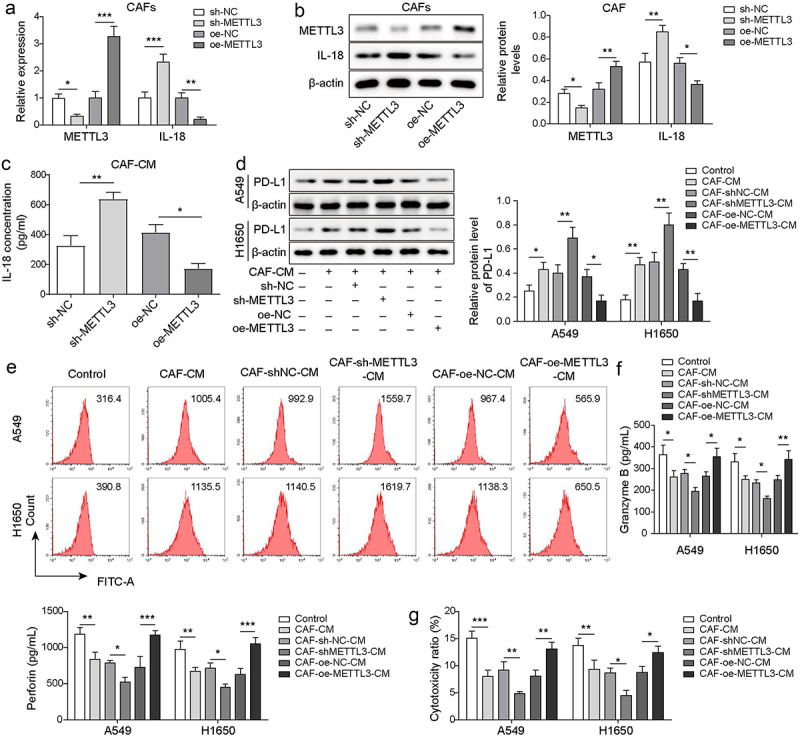
CAF derived METTL3 alleviated PD-L1-mediated immunosuppression of NSCLC through IL-18. CAFs were transfected with sh-METTL3 or oe-METTL3, CAFs that transfected with sh-NC or oe-NC served as the negative control. a-b, the levels of METTL3 and IL-18 in CAFs were detected by qRT-PCR and Western blot. c, the levels of IL-18 in CM from CAFs were detected by ELISA. d-e, PD-L1 levels in A549 and H1650 cells were assessed by Western blot and FCM. f, the levels of granzyme B and perforin in CD8^+^ T cells were detected by ELISA. g, the cytotoxicity of CD8^+^ T cells was detected by using LDH kit. All data were shown as mean ± SD. *n* = 3 per group. *P < 0.05, **P < 0.01, ***P < 0.001.

PD-L1 has been shown to help cancer cells escape immune surveillance by regulating immunosuppression [9]. To explore the effect of m^6^A writers METTL3 on PD-L1-mediated immunosuppression of NSCLC, the levels of METTL3 and IL-18 in CAFs were detected, after METTL3 knockdown or overexpression. Results indicated that METTL3 knockdown significantly up-regulated IL-18 expression in CAFs, while IL-18 expression was significantly inhibited by METTL3 overexpression (Figure 2a,b), indicting IL-18 was negatively regulated by METTL3 in CAFs. Besides, ELISA analysis indicated that METTL3 knockdown increase the secretion of IL-18, but METTL3 overexpression decreased the secretion of IL-18 in CAFs (Figure 2c). Besides, Western blot analysis indicated that compared with CAFs after transfected with shNC, IL-18 was up-regulated in CAFs after transfected with shMETTL3. While after further transfection of oe-METTL3, the up-regulated level of IL-18 was reversed (Figure S1A and B). As showing in Figure 2d,e, PD-L1 was up-regulated in NSCLC cells (A549 and H1650) which were co-cultured with CAFs-CM, and METTL3-knockdown in CAFs accelerated this effect, while overexpression of -METTL3 in CAFs alleviated this effect. These findings suggested that METTL3-knockdown in CAFs negatively regulated PD-L1 expression in NSCLC cells. It is well known that the cytotoxicity of CD8^+^ T cells depends on the secretion of granzyme and perforin [28]. Findings revealed that levels of granzyme B and perforin were decreased in CD8^+^ T cells which were co-cultured with CAF-CM cultured-A549 and H1650 cells, and knockdown of METTL3 in CAFs accelerated this trend, whereas this decreasing trend was rescued by CAFs with METTL3 overexpression (Figure 2f). Consistently, similar results were revealed by statistical analysis of cytotoxicity (Figure 2g). These findings revealed that the cytotoxicity of CD8^+^ T cells was decreased by A549 and H1650 cells which were cocultured with CAF-CM, whereas CAFs with METTL3 overexpression alleviated the inhibitory effect. These findings suggested that METTL3-overexpression in CAFs alleviated immunosuppression of NSCLC.

### METTL3 in CAFs affect IL-18, which in turn affects PD-L1-mediated immunosuppression of NSCLC

To further verify the above experimental results, shMETTL3 and shIL-18 were transfected into CAFs for METTL3 and IL-18 knockdown. qRT-PCR and Western blot analysis showed that compared with shNC group, IL-18 was significantly up-regulated in CAFs after transfected with shMETTL3, but IL-18 knockdown reversed the effect of METTL3 knockdown in CAFs (Figure S2A-B). Similarly, ELISA analysis showed that compared with that shNC group, IL-18 was significantly up-regulated in CAF-CM after transfected with shMETTL3, whereas the effect of METTL3 knockdown was reversed by IL-18 knockdown (Figure S2C). Subsequently, the CM from CAFs which were transfected with shMETTL3, or shMETTL3 and shIL-18 together, were co-cultured with NSCLC cells including A549 and H1650. Western blot analysis showed that compared with that CAF-CM group, PD-L1 was significantly up-regulated in CAF-shMETTL3-CM group, but this trend was alleviated by IL-18 knockdown (Figure S2D-E). Furtherly, A549 and H1650 cells which were co-cultured with CM from CAFs transfected with shMETTL3, or shMETTL3 and shIL-18 together were furtherly co-cultured with CD8^+^ T cells. As showing in Figure S2F and G, the levels of granzyme B and perforin secreted by CD8^+^ T cells which were co-cultured with METTL3 knockdown-CAF-CM were decreased, while this decreasing trend was rescued IL-18 knockdown. Consistently, similar results were revealed by the analysis of cytotoxicity.

### IL-18 served as a directly target of METTL3 to regulate YTHDF2-IL-18 axis in a m^6^A-dependent manner

**Figure 3.**
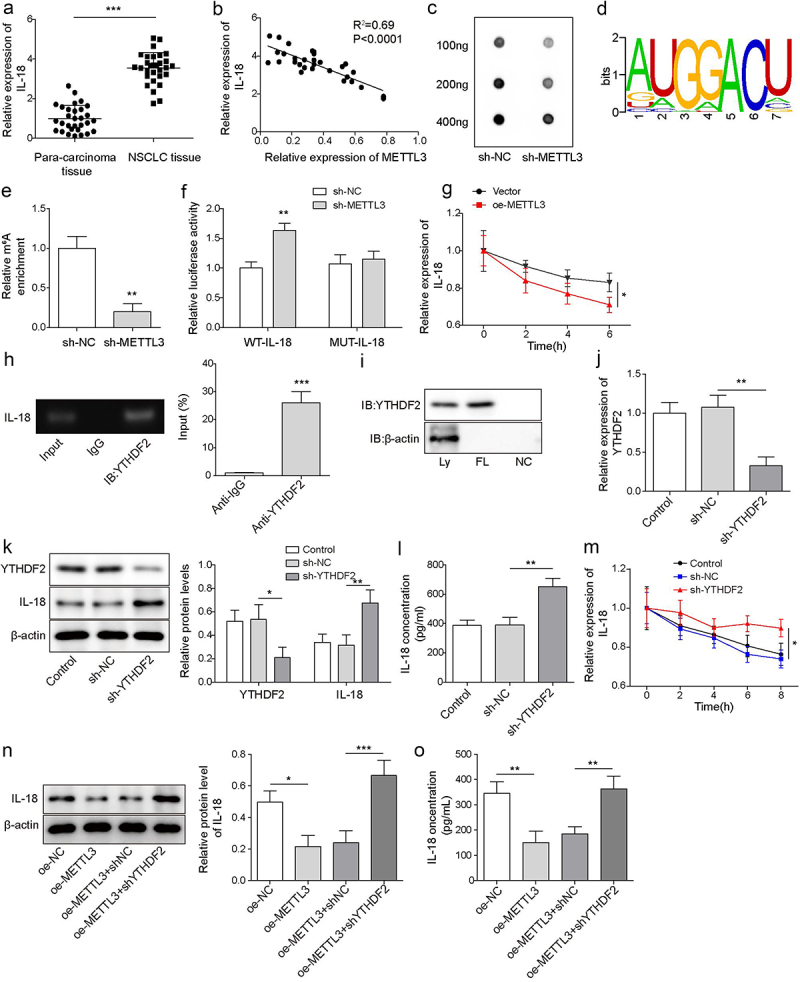
IL-18 was a directly target of METTL3. a, IL-18 mRNA level was detected by qRT-PCR. b, correlation analysis of METTL3 and IL-18 in NSCLC tissues. c, RNA m^6^A dot blot assay was carried to detect the level of RNA m^6^A. d, the m^6^A motif sites of METTL3 coding region were predicted by RMBase v2.0 website. e, MeRIP-qPCR was carried to assess the level of IL-18 m^6^A. f, the direct binding relationship between METTL3 and IL-18 was confirmed by dual-luciferase reporter assay. g, IL-18 mRNA level was detected by qRT-PCR following METTL3 overexpression and actinomycin D treatment. h, the combination of YTHDF2 and IL-18 mRNA was detected by RIP assay. i, the combination of YTHDF2 and IL-18 mRNA was confirmed by RNA pulldown. CAFs were transfected with sh-YTHDF2, CAFs that transfected with sh-NC were served as the negative control. j, YTHDF2 level was detected by RT-qPCR. k, the level of YTHDF2 and IL-18 was measured by Western blot. l, the level of IL-18 was detected by ELISA. m, YTHDF2 knockdown-CAFs were treated with actinomycin D, and the degradation of IL-18 mRNA was evaluated by qRT-PCR. METTL3 overexpressed-CAFs were furtherly transfected with sh-YTHDF2. n, IL-18 was measured by Western blot. o, the level of IL-18 was detected by ELISA. All data were shown as mean ± SD. *n* = 3 per group. *P < 0.01, **P < 0.01, ***P < 0.001.

METTL3 is one of m^6^A writers, which targets pre-mRNAs and various non-coding RNAs, and IL-18 is a tropical cytokine involved in the regulation of immune response [29]. Firstly, we analysed IL-18 expression in NSCLC tissues and para-carcinoma tissues. Results showed that IL-18 was significantly increased in NSCLC tissues (Figure 3a). As showing in Figure 3b, there was a negative correlation between METTL3 and IL-18 in NSCLC (Figure 3b). Subsequently, RNA m^6^A dot blot assay analysis indicated that METTL3-knockdown in CAFs significantly decreased the level of RNA m^6^A (Figure 3c). The m^6^A motif sites of METTL3 coding region were furtherly predicted by RMBase v2.0 website (https://rna.sysu.edu.cn/rmbase/) (Figure 3d). As shown in (Figure 3e,f) the level of IL-18 mRNA m^6^A was significantly inhibited by METTL3 knockdown, and dual-luciferase reporter assay analysis revealed that the luciferase activity of IL-18-WT reported gene was increased by co-transfection of sh-METTL3, but the co-transfection of sh-METTL3 did not affect the luciferase activity of IL-18-MUT reported gene. These findings indicated that IL-18 was a vital target of METTL3 in CAFs.r. As showing in Figure 3g, overexpressed METTL3 in CAFs promoted IL-18 mRNA degradation. All above findings suggested that METTL3 m^6^A methylation modifies IL-18. Besides, RIP assay indicated that YTHDF2 bound to IL-18 mRNA (Figure 3h), and the relationship between YTHDF2 and IL-18 mRNA was confirmed by RNA pulldown assay (Figure 3i). Next, CAFs were transfected with sh-YTHDF2 for YTHDF2 knockdown. qRT-PCR and Western blot analysis showed that YTHDF2 was significantly down-regulated after transfected with sh-YTHDF2 (Figure 3j,k), and the levels of IL-18 were significantly up-regulated after transfected with sh-YTHDF2 (Figure 3k). Similarly, ELISA analysis indicated that the level of IL-18 was significantly up-regulated in the supernatant of CAFs which transfected with sh-YTHDF2 (Figure 3l). Besides, YTHDF2 knockdown inhibited the degradation of IL-18 mRNA (Figure 3m), indicting YTHDF2 promoted IL-18 degradation. Functionally, METTL3 overexpression down-regulated IL-18 level, but YTHDF2 knockdown reversed the downward trend (Figure 3n,o). In total, we suggested that IL-18 served as a directly target of METTL3 to regulated YTHDF2-IL-18 axis in a m^6^A-dependent manner.

### IL-18 was main effector of METTL3 in CAFs against immunosuppression of NSCLC

**Figure 4.**
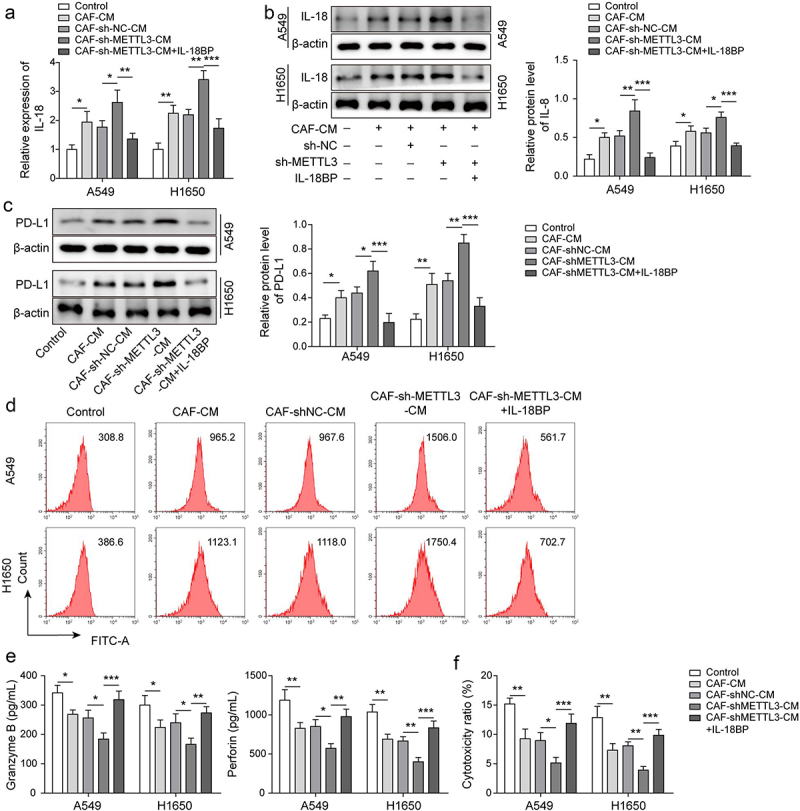
IL-18 was main effector of CAF-derived METTL3 against immunosuppression of NSCLC. a-b, IL-18 level was detected by qRT-PCR and Western blot. c-d, PD-L1 level was assessed by Western blot and FCM. e, the levels of granzyme B and perforin were detected by ELISA. F, the cytotoxicity of CD8^+^ T cells was detected by using LDH kit. All data were shown as mean ± SD. *n* = 3 per group. *P < 0.05, **P < 0.01, ***P < 0.001.

To further explore the effect of METTL3/IL-18 axis on immunosuppression of NSCLC, as showing in Figure 4a,b, IL-18 was highly expressed in A549 and H1650 cells which were co-cultured with CAF-CM, and METTL3-knockdown in CAFs enhanced the facilitation, but IL-18 binding protein (IL-18BP) antagonized the effect of METTL3-knockdown in CAFs IL-18 binding protein (IL-18BP) specifically interacts with IL-18, thereby playing a negative regulatory biological activity of IL-18 [30]. Similarly, PD-L1 expression was upregulated in CAF-CM co-cultured A549 and H1650 cells, and the up-regulated trend was accelerated by coculture with CM from METTL3-knockdown CAFs but the facilitation was antagonized by IL-18 BP (Figure 4c,d). Subsequently, we found that the levels of granzyme B and perforin secreted by CD8^+^ T cells which were cultured with CAF CM-co-cultured A549 and H1650 cells were decreased, and the downward trend was aggravated by CM-derived CAFs with METTL3 knockdown, whereas IL-18BP alleviated the effect (Figure 4e). Furthermore, the cytotoxicity of CD8^+^T cells which were cultured with CAF-CM co-cultured A549 and H1650 cells were decreased, and CM-derived CAFs with METTL3 knockdown exacerbated the downward trend, while the effect of CM-derived CAFs with METTL3 knockdown was alleviated by IL-18BP (Figure 4f). These findings suggested that IL-18 was main effector of METTL3 in CAFs against immunosuppression of NSCLC.

### IL-18 accelerated immunosuppression of NSCLC by driving NF-κB pathway

**Figure 5.**
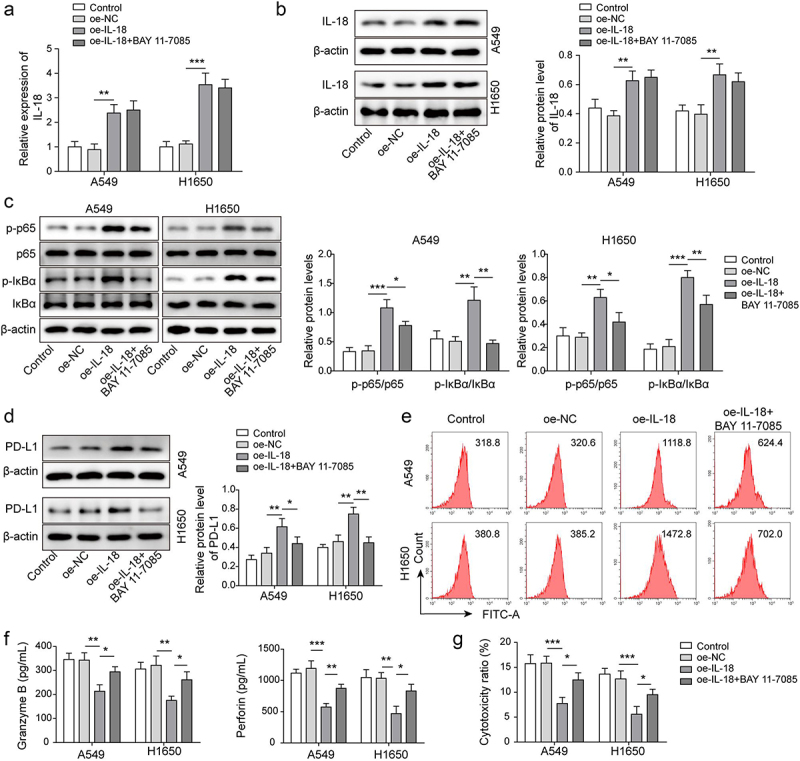
IL-18 accelerated immunosuppression of NSCLC by driving NF-κB pathway. A-B, IL-18 level was detected by qRT-PCR and Western blot. C, NF-κB pathway related proteins’ expression was measured by Western blot. D-E, PD-L1 level was assessed by Western blot and FCM. F, the levels of granzyme B and perforin were detected by ELISA. G, the cytotoxicity of CD8^+^ T cells was detected by using LDH kit. All data were shown as mean ± SD. *n* = 3 per group. *P < 0.05, **P < 0.01, ***P < 0.001.

To further explore the role of IL-18 in immunosuppression of NSCLC, pcDNA3.1-IL-18 plasmids were transfected A549 and H1650 cells for IL-18 overexpression. As showing in Figure 5a,b, IL-18 was significantly up-regulated in A549 and H1650 cells after pcDNA3.1-IL-18 plasmids transfection, and BAY11–7085 had no effect on IL-18 expression. BAY11–7085 serves as an inhibitor of NF-κB pathway. We further analysed the expression of NF-κB pathway related proteins, results showed that the levels of p-p65 and p-IκBα proteins were significantly up-regulated by IL-18 overexpression, while BAY11–7085 antagonized the upward trend (Figure 5c). It has been reported that p65 binds to the CD274 promoter and promotes PD-L1 expression [23]. Consistently, IL-18 overexpression accelerated PD-L1 expression, but which was antagonized by BAY11–7085 (Figure 5d,e). Furthermore, as showing in Figure 5f, the levels of granzyme B and perforin secreted by CD8^+^ T cells which were cultured with IL-18 overexpressed-A549 and H1650 cells were decreased, whereas BAY11–7085 rescued the downward trend. Similarly, the cytotoxicity of CD8^+^T cells which were cultured with IL-18 overexpressed-A549 and H1650 cells were down-regulated, but the decrease trend was rescued by BAY11–7085 (Figure 5g). These findings suggested that IL-18 accelerated immunosuppression of NSCLC by driving NF-κB pathway.

### Knockdown of METTL3 in CAFs accelerated immunosuppression of NSCL *in* *vivo*

**Figure 6.**
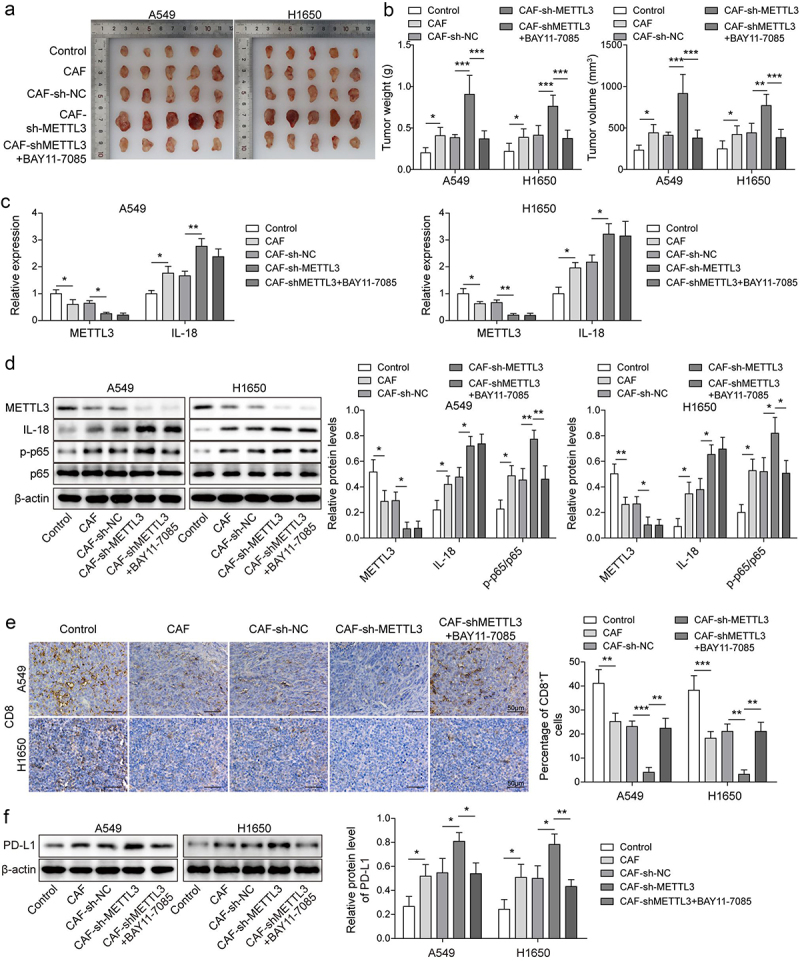
The down-regulation of CAF derived METTL3 accelerated immunosuppression of NSCL. a-b, Statistical analysis of tumour volume and weight of NSCLC mice. c, the levels of METTL3 and IL-18 were detected by qRT-PCR. d, the levels of METTL3, IL-18, p65 and p-p65 were detected by Western blot. e, the distribution of CD8 was detected by IHC, Scale bar = 50 μm. f, the PD-L1 expression status in tumours by western blot. All data were shown as mean ± SD. *n* = 5 per group. *P < 0.05, **P < 0.01, ***P < 0.001.

For further explore the effect of METTL3 in CAFs on immunosuppression of NSCL *in vivo*, A heterotopic implantation model of NSCLC was established in NOD-SCID mice. Statistical analysis showed that the tumour volume and weight of NSCLC mice were significantly increased in CAF, CAFs-shNC group, and CAFs-shMETTL3 accelerated facilitation, after further treatment with BAY11–7085 (an inhibitor of the NF-κB pathway), the tumour volume and weight of NSCLC mice were alleviated (Figure 6a,b). As showing in Figure 6c, METTL3 was down-regulated in CAF and CAF-shNC group, and the downward trend was aggravated by CAF-shMETTL3. Inversely, IL-18 expression was increased in CAF and CAF-shNC group, and CAF-shMETTL3 exacerbated the upward trend. After further treatment with BAY11–7085, the levels of METTL3 and IL-18 did not change. Consistently, the up-regulated p-p65 level in CAF and CAF-shNC group was synergistically promoted by CAF-shMETTL3, but after further treatment with BAY11–7085, p-p65 level was decreased (Figure 6d). Furthermore, Results showed that the proportion of CD8^+^T cells were significantly decrease in CAF and CAF-shNC group, and CAF-shMETTL3 exacerbated the downward trend, while after further treatment with BAY11–7085, the decreasing proportion of CD8^+^T cells was rescued. This phenomenon was verified by IHC analysis (Figure 6e). Moreover, as showing in Figure 6f, the level of PD-L1 were significantly increase in CAF and CAF-shNC group, and CAF-shMETTL3 promoted the upward trend, but this trend was reversed by BAY11–7085. These findings suggested that knockdown of METTL3 in CAFs accelerated immunosuppression of NSCLC *in vivo*.

## Discussion

It is well known that lung cancer is still the leading cause of cancer-related death, and NSCLC accounts for about 85% of its incidence [31]. Increasing evidence shows that the high expression of PD-L1 in activated T cells of NSCLC patients will help tumour cells to form immunosuppression, which will bring some challenges to NSCLC treatment [32]. CAFs are the main component of tumour stroma, which accelerates cancer progression by promoting tumour angiogenesis [33]. In this paper, we mainly discussed the specific mechanism of CAFs regulating NSCLC immunosuppression. Our results illustrated that IL-18 served as a main effector of CAF-derived METTL3 against immunosuppression of NSCLC via regulating NF-κB pathway. Our findings point out a new direction for NSCLC treatment.

m^6^A is a large number of modifications found in mRNA, and m^6^A modification in RNAs will affect gene splicing or translation [34]. Most m^6^A modifications in mRNA is catalysed by the complex of METTL3, METTL14 and WTAP [35]. METTL3 binds to most mRNAs containing m^6^A motifs and functions to regulate the recruitment of m^6^A methyltransferase complexes to mRNA targets [36]. Our results showed that METTL3 was low expression in NSCLC tissues and CAFs. Further analysis indicated that low METTL3 in CAFs facilitated PD-L1-mediated immunosuppression of NSCLC. In detail, METTL3 suppressed IL-18, and inhibited IL-18 furtherly affected PD-L1 expression. These changes accelerated the secretion of granzyme B and perforin, and enhanced the cytotoxicity of CD8^+^ T cells. These findings suggest that low expression of METTL3 in CAFs repressed the immune response of CD8^+^ T and aggravated PD-L1-mediated immunosuppression of NSCLC.

IL-18 stimulates the secretion of interferon-γ (IFN-γ) and has multiple effects on endogenous immune system cells. This characteristic may make IL-18 to be a promising candidate to improve anti-tumour efficacy of T cells [37]. Our findings indicated that IL-18 was negatively regulated by METTL3 in CAFs, and there were m^6^A motif sites in IL-18 coding region, indicating METTL3 May regulate IL-18 in an m^6^A dependent manner. YTHDF2, an m^6^A reader, was reported to selectively bind to m^6^A sites to regulate mRNA degradation [35]. There is evidence that YTHDF2 degrades m^6^A modified mRNA by binding to m^6^A site, thus regulating disease progression, and this process is catalysed by METTL3-centred writers [38]. Moreover, m^6^A methyltransferase METTL3 promotes LPS-induced microglia inflammation through the NF-κB signalling pathway [39]. Our results showed that IL-18 was a directly target of METTL3, the regulation of IL-18 level was regulated by METTL3-related m^6^A modification, and YTHDF2 was the m^6^A methylation reader of IL-18. These findings indicate that METTL3 regulates the YTHDF2-IL-18 axis in an m^6^A-dependent manner. Further functional experiments’ analysis showed that IL-18 was main effector in METTL3-knockdown CAFs involved in immunosuppression of NSCLC.

NF-κB transcription factor plays a key role in the process of immune system, innate immunity and adaptive immune response [40], and NF-κB is the key regulator of T cell proliferation and differentiation [41]. Increasing evidence shows that NF-κB is known to regulate the expression of PD-L1 (CD274 gene) in various diseases [42]. Our results showed that IL-18 activated the NF-KB pathway, which in turn promoted PD-L1 expression. In a word, IL-18 accelerated immunosuppression of NSCLC by driving NF-κB pathway.

Summary, IL-18 served as a main effector of METTL3 in CAFs involved in immunosuppression of NSCLC via driving NF-κB pathway. Our findings point out a new direction for NSCLC treatment.

## Ethics approval and consent to participate

This study was approved by the Ethics Committee of Hunan Cancer Hospital and obtained the written informed consent of all patients. All the experiments were carried out according to approved guidelines.

## Supplementary Material

Supplemental Material

## Data Availability

The raw data supporting the conclusions of this manuscript will be made available by the authors, without undue reservation, to any qualified researcher.
