# Supplementary material for: IL-18 serves as a main effector of CAF-derived METTL3 against immunosuppression of NSCLC via driving NF-κB pathway
Source: Epigenetics. 2023 Oct 23;18(1):2265625. doi: 10.1080/15592294.2023.2265625 (PMC10595399; doi:10.1080/15592294.2023.2265625)
Supplement: Supplemental Material [file KEPI_A_2265625_SM5028.zip › Supplementary files/Supplementary caption.docx]

**Figure S1 METTL3 inhibits the level of IL-18 in CAFs.** A, shMETTL3 was transfected into CAFs for METTL3 knockdown, sh-NC served as the negative control. Then, METTL3 knockdown-CAFs were furtherly transfected with oe-METTL3. Western blot measured the levels of METTL3 and IL-18. B, the level of IL-18 in CM from CAFs was detected by ELISA. All data were shown as mean ± SD. n =3 per group. **P* < 0.01, ** *P* < 0.01.

**Figure S2 CAFs affect IL-18 by affecting METTL3, which in turn affects PD-L1-mediated immunosuppression of NSCLC**. shMETTL3 and shIL-18 were transfected into CAFs for METTL3 and IL-18 knockdown. A-B, qRT-PCR and Western blot were performed to detected the level of IL-18 in CAFs. C, ELISA was performed to assess the level of IL-18 in CAF-CM. Subsequently, CM from CAFs transfected with shMETTL3 alone, shMETTL3 and shIL-18 together, were co-cultured with NSCLC cells including A549 and H1650. D-E, Western blot and FCM were performed to measure the level of PD-L1, respectively. Furtherly, A549 and H1650 cells which were co-cultured with CM from CAFs transfected with shMETTL3 alone, shMETTL3 and shIL-18 together were furtherly were co-cultured with CD8^+^ T cells. F-G, the levels of granzyme B and perforin were detected by ELISA, and the cytotoxicity of CD8^+^ T cells was detected by using LDH kit. All data were shown as mean ± SD. n =3 per group. **P* < 0.01, ** *P* < 0.01, *** *P* < 0.001.
