# Supplementary figures and images for: IL-18 serves as a main effector of CAF-derived METTL3 against immunosuppression of NSCLC via driving NF-κB pathway
Source: Epigenetics. 2023 Oct 23;18(1):2265625. doi: 10.1080/15592294.2023.2265625 (PMC10595399; doi:10.1080/15592294.2023.2265625)

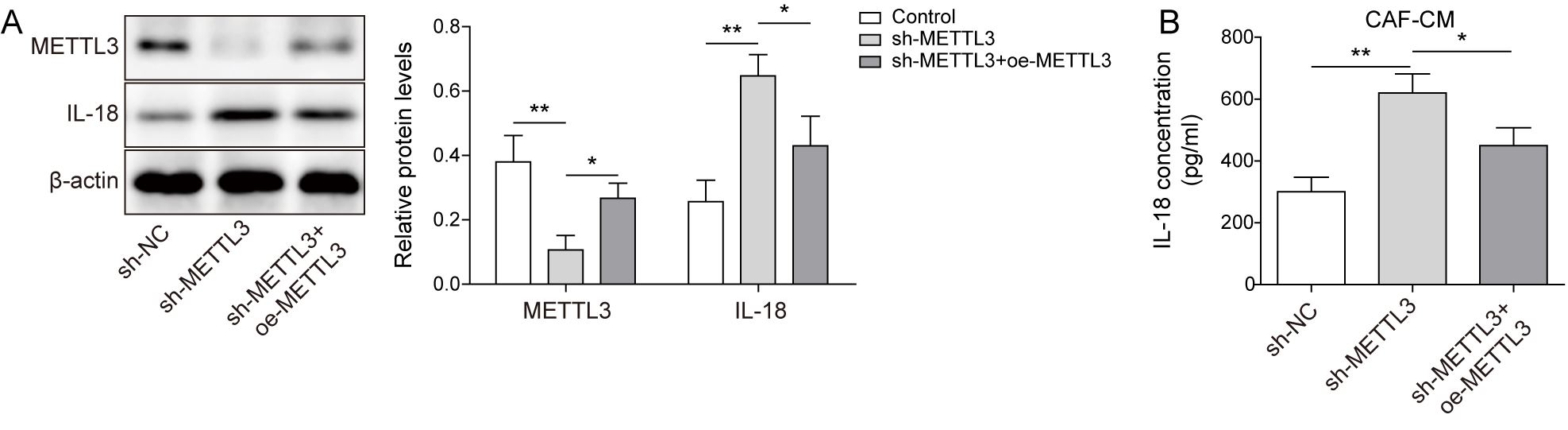

Supplement: Supplemental Material [file KEPI_A_2265625_SM5028.zip › Supplementary files/figS1.jpg]

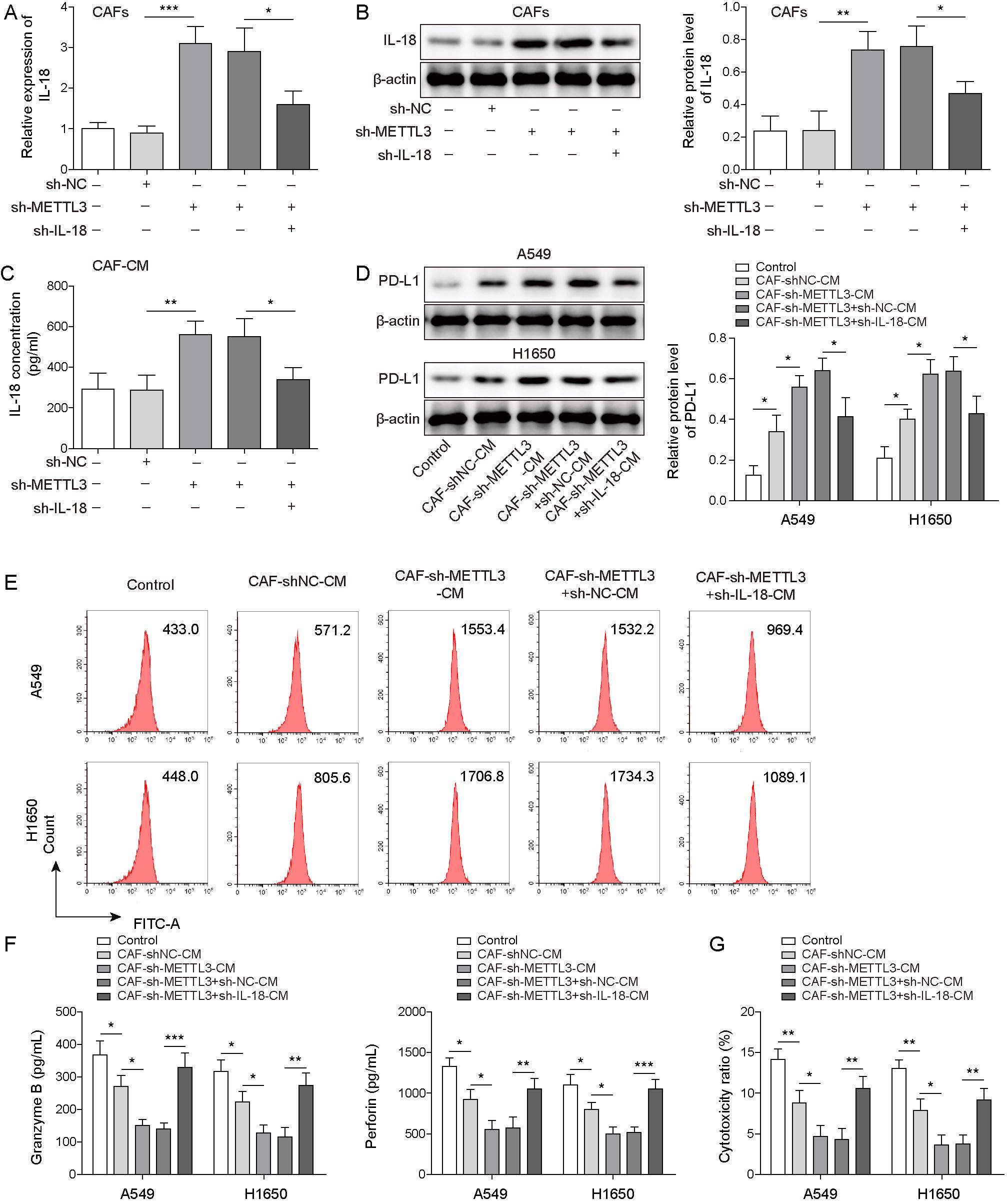

Supplement: Supplemental Material [file KEPI_A_2265625_SM5028.zip › Supplementary files/figS2.jpg]
